# Transdisciplinary treatment of Class III malocclusion using conventional implant-supported anchorage: 10-year posttreatment follow-up

**DOI:** 10.1590/2176-9451.20.3.069-079.oar

**Published:** 2015

**Authors:** Mariana Roennau Lemos Rinaldi, Susana Maria Deon Rizzatto, Luciane Macedo de Menezes, Waldemar Daudt Polido, Eduardo Martinelli Santayanna de Lima

**Affiliations:** 1 PhD resident in Orthodontics, Pontifícia Universidade Católica do Rio Grande do Sul (PUCRS), Porto Alegre, Rio Grande do Sul, Brazil; 2 Professor of Orthodontics, Pontifícia Universidade Católica do Rio Grande do Sul (PUCRS), Porto Alegre, Rio Grande do Sul, Brazil; 3 PhD in Oral and Maxillofacial Surgery, Pontifícia Universidade Católica do Rio Grande do Sul (PUCRS), Porto Alegre, Rio Grande do Sul, Brazil; 4 Adjunct professor of Orthodontics, Pontifícia Universidade Católica do Rio Grande do Sul (PUCRS), Porto Alegre, Rio Grande do Sul, Brazil

**Keywords:** Orthodontic anchorage procedures, Dental implants, Angle Class III malocclusion, Tooth loss

## Abstract

**INTRODUCTION:**

Combined treatment offers advantages for partially edentulous patients. Conventional implants, used as orthodontic anchorage, enable previous orthodontic movement, which provides appropriate space gain for crown insertion.

**OBJECTIVE:**

This case report describes the treatment of a 61-year and 10-month-old patient with negative overjet which made ideal prosthetic rehabilitation impossible, thereby hindering dental and facial esthetics.

**CASE REPORT:**

After a diagnostic setup, conventional implants were placed in the upper arch to anchor intrusion and retract anterior teeth. Space gain for lateral incisors was achieved in the lower arch by means of an orthodontic appliance.

**CONCLUSIONS:**

Integrated planning combining Orthodontics and Implantology provided successful treatment by means of conventional implant-supported anchorage. The resulting occlusal relationship proved stable after 10 years.

## Abstract (pt)

**INTRODUÇÃO:**

tratamentos combinados podem oferecer vantagens em casos de edentulismo parcial. O uso de implantes convencionais como ancoragem ortodôntica permite a movimentação ortodôntica prévia, fornecendo os espaços apropriados para inserção das coroas.

**OBJETIVO:**

este relato de caso descreve o tratamento de uma paciente, com 61 anos e 10 meses de idade, que possuía um *overjet* negativo que tornava impossível uma reabilitação protética ideal, comprometendo a estética dentária e facial.

**RELATO DO CASO:**

após um *setup* diagnóstico, os implantes convencionais foram colocados na mandíbula para ancorar a intrusão e retração dos dentes anteriores. Espaços para os incisivos laterais foram abertos na maxila, usando-se aparelho ortodôntico.

**CONCLUSÕES:**

o planejamento integrado entre Ortodontia e Implantodontia propiciou um tratamento bem-sucedido, usando implantes convencionais como ancoragem. A relação oclusal obtida apresenta estabilidade 10 anos pós-tratamento.

## INTRODUCTION

Transdisciplinarity is a trend in Dentistry as well as in other areas of the health sciences. This is because the interaction established among different specialties provides patients with a comprehensive treatment plan.^1^^,^ 2 ^,^ 3 Osseointegration has opened up new possibilities not only for Prosthodontics, but also for Orthodontics. Proper anchorage has always been fundamental for orthodontic treatment efficiency, as it allows the desired orthodontic movements to be performed and reduces potential adverse effects. The use of conventional implants and temporary anchorage devices (TAD) has improved anchorage control and provided absolute resistance units against movement. Absolute anchorage allows space closure, intrusion, extrusion, protraction, retraction of teeth and stabilization of periodontally compromised teeth.^4^^-^ 8

Conventional implants for prosthetic restoration can also be used for orthodontic anchorage.^9^ Implant selection and insertion site should be appropriate for the dual function of implants: rehabilitation and anchorage. The anatomical aspects of the case, the intended orthodontic movement and the ideal position for final rehabilitation should be planned ahead of time.^5^^,^ 10 ^,^ 11 Combined treatment offers advantages for partially edentulous patients, and so does previous orthodontic movement, as it provides appropriate space gain for implant insertion.^12^^-^ 15

The aim of this case report is to demonstrate the transdisciplinary treatment of a Class III malocclusion patient with multiple missing teeth. Conventional implants were used as anchorage to retract lower teeth. This combined transdisciplinary plan intended to maximize patient's benefits, enhance dental esthetics and establish a balanced occlusion associated with healthy tissues. This report illustrates a case of successful 10-year posttreatment stability.

## CASE REPORT

**Figure 1.**
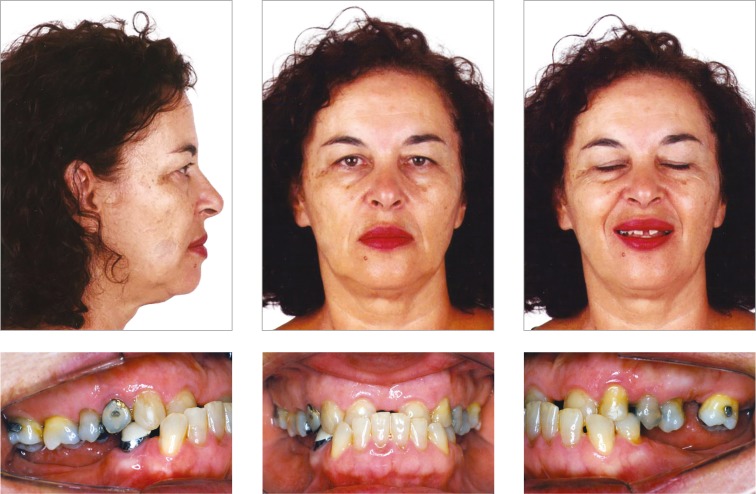
Pre-treatment facial and intraoral photographs.

**Figure 2.**
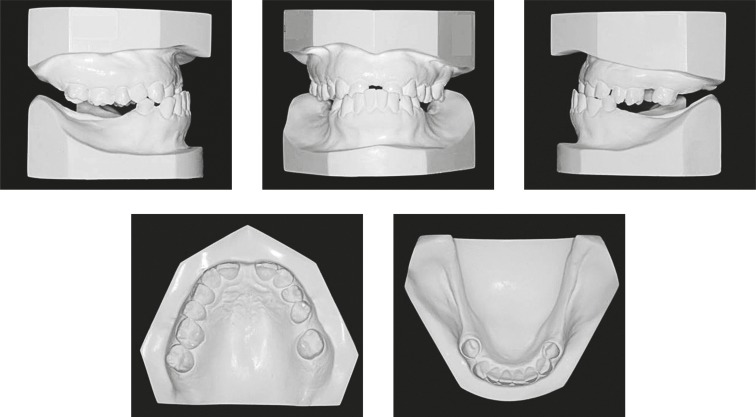
Initial dental casts.

In 1998, a healthy female patient, aged 61 years and 10 months old, presented at the orthodontic service of the Brazilian Dental Association with anterior crossbite and multiple missing teeth. Her chief complaint was related to poor dental esthetics. Prosthetic rehabilitation was thought to be determined by the conditions of dental occlusion. There was premature contact between lower central incisors, and anterior crossbite was mostly caused by functional sliding resulting from contact. In occlusion, the patient had Class I canine relationship on both sides (Figs 1 and 2).

Patient's face was symmetrical in frontal view, with a marked nasolabial fold. Facial profile was unbalanced, with mild maxillary deficiency and protrusion of the lower lip which was positioned ahead of the upper lip. The nasolabial angle denoted the incorrect anterior-posterior position of the maxilla, which was confirmed by cephalometric findings. The mentolabial sulcus was flat, most likely due to muscle adaptation to anterior crossbite.

**Figure 3.**
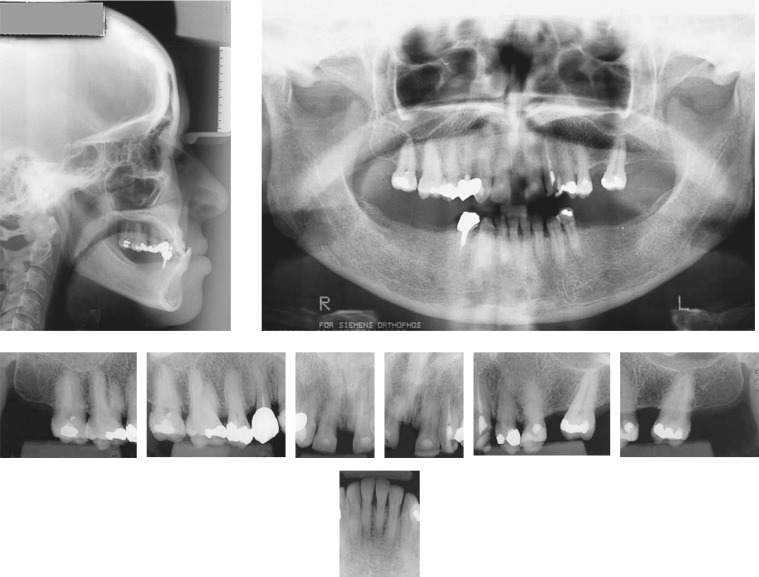
Initial lateral cephalometric radiograph, panoramic and periapical radiographs.

Lower dentition was mutilated: molars and second premolars were absent on both sides. On the right side, there was a single-unit ceramic crown over the lower first premolar. In the upper arch, posterior teeth were extruded, left first molar was absent and right first premolar had a ceramic crown. A midline diastema of 5 mm was found in the upper arch and associated to the migration of central incisors and to the space of missing lateral incisors (Fig 3).

**Figure 4.**
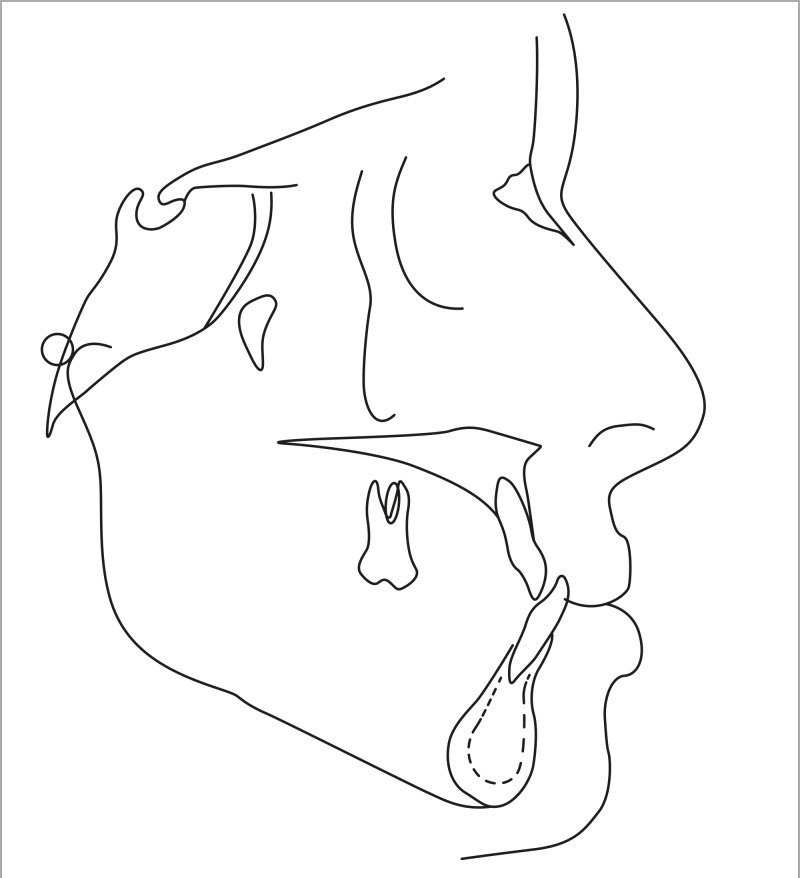
Initial cephalometric tracing.

Periapical radiographs revealed generalized mild attachment loss, which suggested judicious periodontal control during orthodontic treatment. In spite of the edentulous regions, bone height was enough for conventional implant placement. Cephalometric evaluation revealed skeletal Class III malocclusion associated with retrusion of upper incisors and protrusion of lower incisors (Fig 4and Table 1).

**Table 1.** Cephalometric data.

	Pre-treatment	Post-treatment	10-year follow-up
SNA (degrees)	82	81	81
SNB (degrees)	83	81	81.5
ANB (degrees)	-1	0	-0.5
1.NA (degrees)	14	25	25.5
1-NA (mm)	4.0	9	9
1.NB (degrees)	24.5	23	23
1-NB (mm)	8.0	5.5	5.5
Pog-NB (mm)	1.5	0.5	0.6
1:1 (degrees)	142	132	
SN:OP (degrees)	9	12	12.5
SN:GoGn (degrees)	31	32	32
S to upper lip (mm)	-1	1	1
S to lower lip (mm)	2	1	3
FMA (degrees)	28	29.5	29.5
FMIA (degrees)	61	62.5	61.5
IMPA (degrees)	91	88	89
Angle of convexity (degrees)	-4	-2	-2.5

The objectives of treatment were: (1) correct anterior crossbite; (2) reestablish vertical dimensions in the posterior region, which would provide space gain for implant-retained prosthetic restorations in the region of lower premolars and molars; (3) close interincisal diastema; (4) gain space for implants and prosthetic crowns in the region of upper lateral incisors; and (5) improve the relationship established between upper and lower lips.

Delay in rehabilitation treatment after extraction of posterior teeth is expected to provoke alveolar bone atrophy; therefore, only basal bones of the maxilla and mandible remain intact. Lack of dental occlusion in the posterior region leads to a reduction in lower facial height and changes in the position of remaining teeth. The mandible rotates anticlockwise, remodeling the condyle process and the glenoid fossa.^16^^,^ 17 Orthognathic surgery may be the first choice to correct anterior crossbite and provide the height necessary for prosthetic rehabilitation in the posterior region. Presurgical Orthodontics may create space for implants to replace missing lateral incisors.

Surgery was considered a risky procedure for a 61-year-old patient. She presented favorable conditions for camouflage, since adequate anchorage could be provided. Conventional dental implants can also be used for orthodontic anchorage. Upper incisors should be proclined so as to increase arch perimeter, which would help space gain for upper lateral incisors. Treatment plan was designed according to patient's needs and expectations.

**Figure 5.**
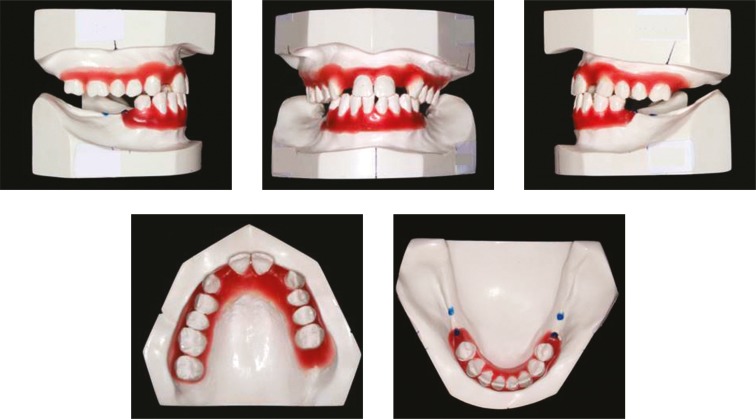
Setup records.

A diagnostic setup was performed according to cephalometric findings. Lower central incisors underwent retrusion of 4 mm and intrusion of 1.5 mm (Fig 5). Upper central incisors were subsequently positioned in contact, with an increase in buccal inclination so as to achieve a 2-mm overjet. Bilateral spaces of 6 mm were created to replace missing upper lateral incisors.

Implants should be inserted 2 mm distal to the lower right first premolar and 3 mm distal to the lower left first premolar. Temporary acrylic crowns were adapted over conventional dental implants (Brånemark System, Nobel Biocare, Kloten, Switzerland: 11.5 x 5 mm in the region of molars and 4 x 11.5 mm in the region of premolars) on both sides of the lower arch. Orthodontic brackets were bonded after six months. Absolute anchorage unit allowed distal movement of lower right canine and left premolar; in addition, it provided retraction and intrusion of lower incisors.

Upper molars were banded and a full fixed orthodontic appliance was placed (Standard Edgewise 0.022 x 0.028-in, 3M-Unitek, Monrovia, USA). Leveling and alignment followed the sequence of stainless steel archwires in increasing stiffness (3M-Unitek, Monrovia, USA). Upper diastema closure and distal movement of lower teeth were performed by sliding mechanics with elastomeric chains.

Retraction of lower incisors and proclination of upper incisors occurred simultaneously. Tear drop loops were bent in 0.018 x 0.025-in stainless steel archwires halfway between lateral incisors and canines. Ideal 0.019 x 0.026-in stainless steel archwires allowed detailed angulation to be performed. Total treatment lasted 36 months.

Maxillary implants were inserted after orthodontic space opening (Brånemark System, Nobel Biocare, Kloten, Switerland: 3.3 x 13 mm on the right side and 3.3 x 15 mm on the left side).

## RESULTS

**Figure 6.**
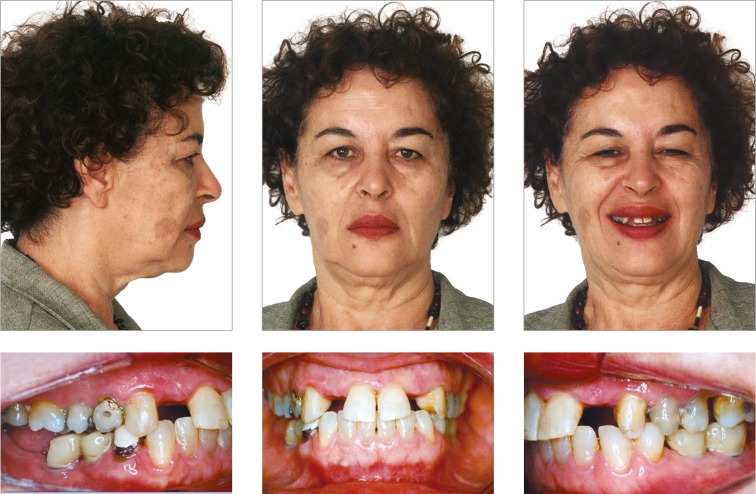
Post-treatment facial and intraoral photographs.

**Figure 7.**
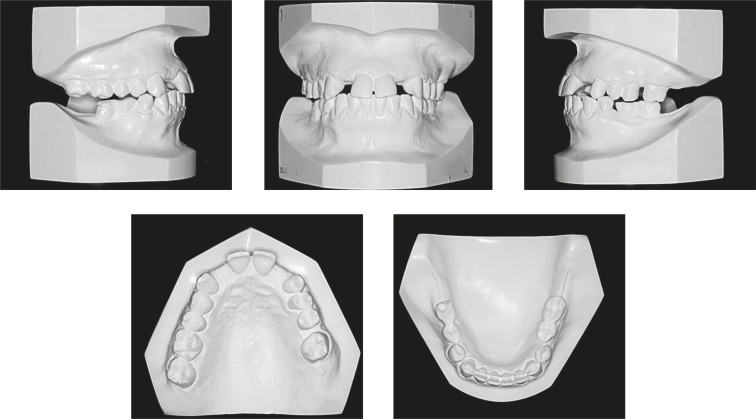
Final dental casts.

**Figure 8.**
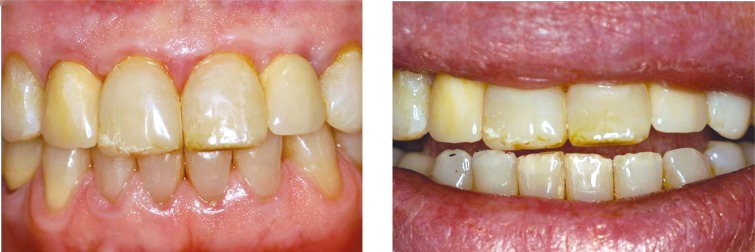
Rehabilitation of the upper incisor region.

By the end of orthodontic treatment, ideal overjet and overbite were achieved. In addition, the necessary space gain for implant-supported definite crowns, placed in the posterior region of the lower arch, was achieved (Figs 6 and 7). Midline upper diastema was closed, which favored prosthetic rehabilitation of lateral incisors. After bracket debonding, definite crowns were placed over implants, central incisors were restored with resin veneers and other damaged restorations were replaced (Fig 8).

**Figure 9.**
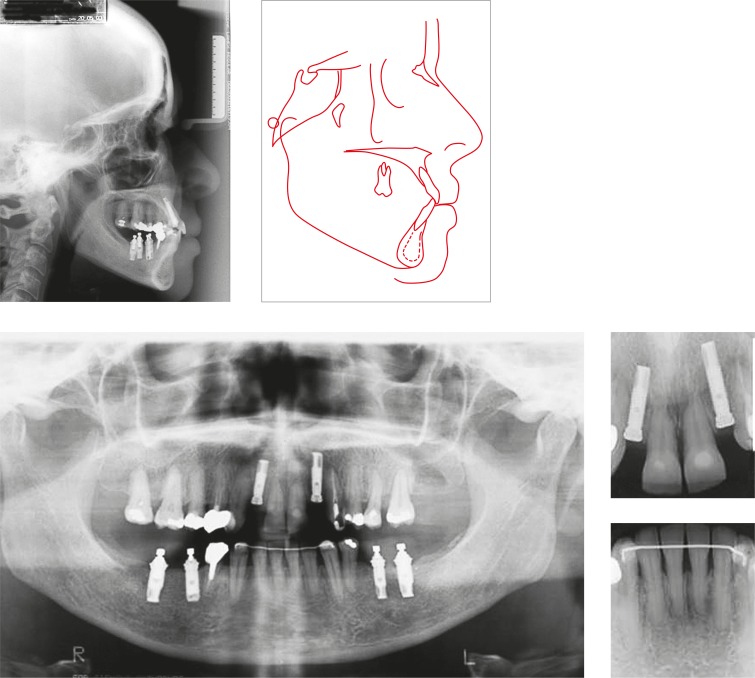
Final lateral cephalometric radiograph, panoramic and periapical radiographs, and cephalometric tracing at treatment completion

**Figure 10.**
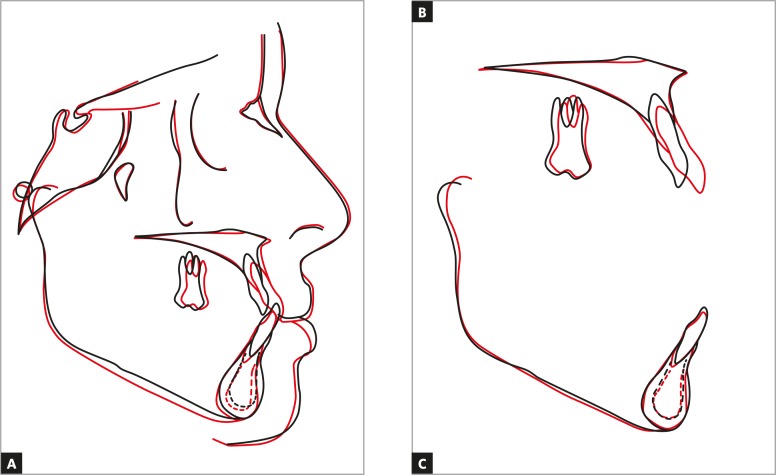
Superimposition of cephalometric tracings at treatment onset (black) and after treatment completion (red): A) Sella-nasion plane at sella; B) Best-fit of the maxilla; C) Mandibular plane at the internal symphysis cortical plate to assess tooth movement, intrusion and incisor repositioning.

Superimposition of cephalometric tracings revealed that the mandible rotated clockwise (FMA from 28° to 29.5°). There was an increase in the occlusal plane angle (SN-OP from 9° to 12°) and a decrease in the incisor-mandible plane angle (IMPA from 91° to 88°), which reflects intrusion and retrusion, respectively, of lower incisors. Proclination of upper incisors was highlighted by an increase in the 1.NA angle (14° to 25°) (Figs 9 and 10, Table 1).

Regarding the facial profile, maxillary deficiency was camouflaged. Upper and lower lips were improved (upper lip to S-line, from -1 to 1 mm; lower lip to S-line, from 2 to 1 mm) with upper incisors support.

**Figure 11.**
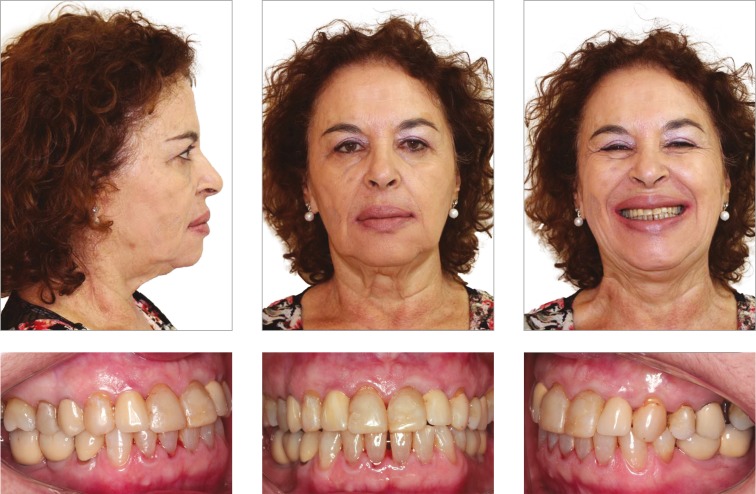
Facial and intraoral photographs 10 years after treatment completion.

**Figure 12.**
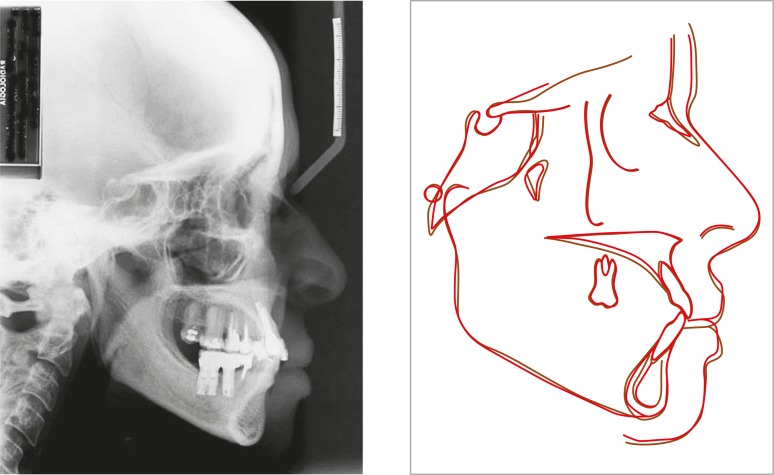
Radiograph 10 years after treatment completion. Superimposition of cephalometric tracings at treatment completion (red) and 10 years after treatment (brown). Sella-nasion plane at sella.

Ten years after the completion of the case, the patient showed occlusal stability, as well as integrity of dentition and prostheses. Resin veneers showed pigmentation and discoloration, as expected. Periodontal structures remained healthy (Figs 11 and 12).

## DISCUSSION

Anterior-posterior and transversal Class III malocclusion relationships tend to worsen with aging.^18^^,^ 19 Patient's Class III skeletal pattern associated with loss of lower posterior teeth were limiting factors in the planning of this case. Without orthognathic surgery, conventional mechanics would not solve the patient's problem. However, there are increased risks associated with surgery and, for this reason, the patient ultimately elected not to undergo surgery.

In this case, prognosis for camouflage was very favorable, considering mild maxillary deficiency and the possibility to procline upper central incisors. The need for oral rehabilitation led this case to be planned based on the use of implants and prostheses. Dentistry restored key features of patient's quality of life: proper mastication as well as smile and facial esthetics. In 1998, the life expectancy for women in Brazil was 72 years.^20^ Thus, we offered a reliable treatment which promotes long-term oral health to our patient. Additionally, transdisciplinary treatment plan fulfilled patient's needs and expectations.

In the late 1990s, skeletal anchorage in Orthodontics was not as usual as it is today. Therefore, we considered the possibility of losing implants with the application of orthodontic movement and occlusal forces, leading to decreased alveolar width and height.

Orthodontic forces are small when compared to the complex system of intermittent and multidirectional forces acting on implants during mastication. Thus, biomechanical responses are within biological limits; for instance, an elastic chain used for canine retraction leads to a force of 1 N or less. The association between orthodontic forces and function stimulates responses of bone modeling and remodeling, which may lead to a new balance of forces.^10^^,^ 16

The approach presented herein took advantage of conventional implants which functioned as orthodontic anchorage before prosthetic procedures.^8^^,^ 21 Therefore, implants placed in the region of lower molars provided anchorage necessary for intrusion and retraction of anterior lower teeth. This was considered a worthwhile strategy: previous orthodontic treatment improved occlusion and created space necessary for crown placement (Fig 6).^8^^,^ 10 ^,^ 14 ^,^ 22 Implant selection and insertion site must consider patient's anatomical features, quality and quantity of bone available (alveolar width and height), gingival conditions, ideal position for teeth replacement and orthodontic movement.^4^^,^ 14 ^,^ 23 ^,^ 24

Whenever anterior teeth are missing, it is challenging to obtain a natural smile and achieve correct occlusion. Before implants were developed, alternative therapies for these cases included the use of adhesive crowns and preparation of healthy teeth to function as pillars. Both treatment options have esthetics limitations.^25^^,^ 26

The esthetic objectives of implant therapy include creating adequate gingival margins without abrupt changes in tissue height, maintaining the papilla intact and preserving alveolar crest convex contour. To this end, 1-mm space or more, between the implant and the adjacent tooth root, is required in addition to adequate space for crown placement.^14^ Whenever it is impossible to gain the space required, space closure with mesial movement of posterior teeth is a reasonable option, especially if only one or two teeth are missing in the anterior region.^13^

No consensus has been reached regarding the best treatment option to replace missing lateral upper incisors.^27^ It is important to consider various aspects of treatment, namely: patient's age, alveolar ridge and gingiva, type of malocclusion, other missing teeth and the possibility to restore space. Implant placement is the best choice for cases similar to that demonstrated in the present report: multiple missing teeth, interincisal diastema and mild Class III malocclusion. Space closure would have caused the collapse of the upper arch, thereby reducing arch perimeter. Mesial movement of central incisors produced the necessary space for implant placement. This was based on the margin of space required to the roots of adjacent natural teeth.^2^^,^ 3 ^,^ 7

In addition to absolute anchorage provided by implants, biomechanics was similar to the conventional technique. This treatment option requires the understanding of forces involved in the system and the ability to control the magnitude of forces on implants. Implants are structures fixed to bone and which transfer the load to the teeth which, in turn, are connected by the appliance. It is important to consider the functional characteristics of occlusion with implants, which assures stability and success (Figs 11 and 12).^17^^,^ 28

## CONCLUSION

The goals of this transdisciplinary treatment were to create adequate space in vertical, transversal and horizontal planes for dental implant and prosthesis placement, with a view to establishing functional occlusion and attractive dentition. Treatment plan combining Implantology, Orthodontics and Prosthodontics proved to be effective in overcoming the challenges. Tissue stability and healthy conditions remain after a 10-year posttreatment follow-up, which confirms the usefulness of this approach.

## References

[1] Agarwal S, Gupta S, Chugh VK, Jain E, Valiathan A, Nanda R (2014). Interdisciplinary treatment of a periodontally compromised adult
patient with multiple missing posterior teeth. Am J Orthod Dentofacial Orthop.

[2] Pinho T, Neves M, Alves C (2012). Multidisciplinary management including periodontics, orthodontics,
implants, and prosthetics for an adult. Am J Orthod Dentofacial Orthop.

[3] Uribe F, Janakiraman N, Nanda R (2013). Interdisciplinary approach for increasing the vertical dimension of
occlusion in an adult patient with several missing teeth. Am J Orthod Dentofacial Orthop.

[4] Shapiro PA, Kokich VG (1988). Uses of implants in orthodontics. Dent Clin North Am.

[5] Nanda R (2005). Biomechanics and esthetic strategies in clinical orthodontics.

[6] Uribe F, Nanda R (2009). Intramaxillary and intermaxillary absolute anchorage with an
endosseous dental implant and rare-earth magnets. Am J Orthod Dentofacial Orthop.

[7] Barros LAB, Almeida Cardoso M, de Avila ÉD, Molon RS, Siqueira DF, Mollo-Junior FA (2013). Six-year follow-up of maxillary anterior rehabilitation with forced
orthodontic extrusion: achieving esthetic excellence with a multidisciplinary
approach. Am J Orthod Dentofacial Orthop.

[8] Kuroda S, Iwata M, Tamamura N, Ganzorig K, Hichijo N, Tomita Y (2014). Interdisciplinary treatment of a nonsyndromic oligodontia patient with
implant-anchored orthodontics. Am J Orthod Dentofacial Orthop.

[9] Alani A, Bishop K, Renton T, Djemal S (2014). Update on guidelines for selecting appropriate patients to receive
treatment with dental implants: priorities for the NHS-the position after 15
years. Br Dent J.

[10] Favero L, Brollo P, Bressan E (2002). Orthodontic anchorage with specific fixtures: related study
analysis. Am J Orthod Dentofacial Orthop.

[11] Chang JZ-C, Liu P-H, Wang Y-T, Chen Y-J, Yao C-CJ, Lai EH-H (2011). Orthodontic-prosthetic implant anchorage in a partially edentulous
patient. J Dent Sci.

[12] Farret MM, Benitez Farret MM (2013). Skeletal Class III malocclusion treated using a non-surgical approach
supplemented with mini-implants: a case report. J Orthod.

[13] Bilodeau JE (2014). A "midline dilemma" in an adult mutilated dentition. Am J Orthod Dentofacial Orthop.

[14] Rose TP, Jivraj S, Chee W (2006). The role of orthodontics in implant dentistry. Br Dent J.

[15] Moslehifard E, Nikzad S, Geraminpanah F, Mahboub F (2012). Full-mouth rehabilitation of a patient with severely worn dentition
and uneven occlusal plane: a clinical report. J Prosthodont.

[16] Higuchi KW (2000). Orthodontic applications of osseointegrated implants.

[17] Melsen B, Lang NP (2001). Biological reactions of alveolar bone to orthodontic loading of oral
implants. Clin Oral Implants Res.

[18] Berg RE, Espeland L, Stenvik A (2008). A 57-year follow-up study of occlusion. Part 3: Oral health and
attitudes to teeth among individuals with crossbite at the age of 8
years. J Orofac Orthop.

[19] Miyajima K, McNamara JA, Sana M, Murata S (1997). An estimation of craniofacial growth in the untreated Class III female
with anterior crossbite. Am J Orthod Dentofacial Orthop.

[20] IBGE. Coordenação de População e Indicadores Sociais (2008). Gerência de estudos e análises da dinâmica demográfica projeção da população
do Brasil por sexo e idade para o período 1980-2050 revisão 2008.

[21] Martin W, Heffernan M, Ruskin J (2002). Template fabrication for a midpalatal orthodontic implant: technical
note. Int J Oral Maxillofac Implants.

[22] Smalley WM (1995). Implants for tooth movement: determining implant location and
orientation. J Esthet Dent.

[23] Odman J, Lekholm U, Jemt T, Branemark PI, Thilander B (1988). Osseointegrated titanium implants-a new approach in orthodontic
treatment. Eur J Orthod.

[24] Thilander B, Odman J, Lekholm U (2001). Orthodontic aspects of the use of oral implants in adolescents: a
10-year follow-up study. Eur J Orthod.

[25] Zachrisson BU (1978). Improving orthodontic results in cases with maxillary incisors
missing. Am J Orthod.

[26] Czochrowska EM, Skaare AB, Stenvik A, Zachrisson BU (2003). Outcome of orthodontic space closure with a missing maxillary central
incisor. Am J Orthod Dentofacial Orthop.

[27] Andrade D, Loureiro C, Araújo V, Riera R, Atallah A (2013). Treatment for agenesis of maxillary lateral incisors: a systematic
review. Orthod Craniofac Res.

[28] Southard TE, Buckley MJ, Spivey JD, Krizan KE, Casko JS (1995). Intrusion anchorage potential of teeth versus rigid endosseous
implants: a clinical and radiographic evaluation. Am J Orthod Dentofacial Orthop.

